# An Actuated Variable-View Rigid Scope System to Assist Visualization in Diagnostic Procedures

**DOI:** 10.1109/JTEHM.2024.3407951

**Published:** 2024-05-31

**Authors:** Sofia Basha, Mohammad Khorasani, Nihal Abdurahiman, Jhasketan Padhan, Victor Baez, Abdulla Al-Ansari, Panagiotis Tsiamyrtzis, Aaron T. Becker, Nikhil V. Navkar

**Affiliations:** Department of SurgeryHamad Medical Corporation Doha Qatar; Electrical and Computer EngineeringUniversity of Houston 14743 Houston TX 77004 USA; Department of Mechanical EngineeringPolitecnico di Milano Milan 20133 Italy; Department of StatisticsAthens University of Economics and Business 59164 Athens 104 34 Greece

**Keywords:** Articulated scope, diagnosis, endoscopy, rigid scope, scope holder

## Abstract

Objective: Variable-view rigid scopes offer advantages compared to traditional angled laparoscopes for examining a diagnostic site. However, altering the scope’s view requires a high level of dexterity and understanding of spatial orientation. This requires an intuitive mechanism to allow an operator to easily understand the anatomical surroundings and smoothly adjust the scope’s focus during diagnosis. To address this challenge, the objective of this work is to develop a mechanized arm that assists in visualization using variable-view rigid scopes during diagnostic procedures.Methods: A system with a mechanized arm to maneuver a variable-view rigid scope (EndoCAMeleon - Karl Storz) was developed. A user study was conducted to assess the ability of the proposed mechanized arm for diagnosis in a preclinical navigation task and a simulated cystoscopy procedure.Results: The mechanized arm performed significantly better than direct maneuvering of the rigid scope. In the preclinical navigation task, it reduced the percentage of time the scope’s focus shifted outside a predefined track. Similarly, for simulated cystoscopy procedure, it reduced the duration and the perceived workload.Conclusion: The proposed mechanized arm enhances the operator’s ability to accurately maneuver a variable-view rigid scope and reduces the effort in performing diagnostic procedures.Clinical and Translational Impact Statement: The preclinical research introduces a mechanized arm to intuitively maneuver a variable-view rigid scope during diagnostic procedures, while minimizing the mental and physical workload to the operator.

## I. Introduction

Rigid scopes are primarily used during a minimally invasive surgical procedure to visualize the operative field [1]. In addition, they are also used for diagnostic procedures. During diagnosis, the rigid scope is introduced into the body through a natural opening and directed to the specific area of interest. The scope is methodically advanced along a narrow passageway and maneuvered to examine the tissue linings, identify any abnormal growths such as polyps, and determine the underlying cause of issues. While a flexible endoscope can be also be used, a rigid scope offers distinct advantages, such as superior optical resolution, ease in sterilization, ease in maintenance, and lower cost [2], [3], [4]. This has led to wider adoption of the rigid scope in diagnostic procedures such as rhinoscopy [5], cystoscopy [6], and transvaginal endoscopy [7].

Traditional rigid scopes utilized in clinical settings are available in various sizes, made up of different diameters, shaft lengths, and viewing angles to accommodate a wide range of diagnostic or therapeutic procedures. In terms of the viewing angle, these scopes are categorized as forward viewing (0°), oblique viewing (12°, 30°, 45°), lateral viewing (70° or 90°), or retrograde viewing (120°). Since rigid scopes have a fixed viewing angle with limited field-of-view, and the shaft lacks effective maneuverability in tight and confined spaces, variable viewing-angle rigid scopes (also known as articulated scopes) were developed. Examples include EndoCAMeleon - Karl Storz and Endorizon Sinuscope - Olympus. These scopes enable variable viewing direction in one plane, and when combined with 360° rotation of the scope shaft along its axis, increases the overall field of view [2], [3], [8].

**FIGURE 1.**
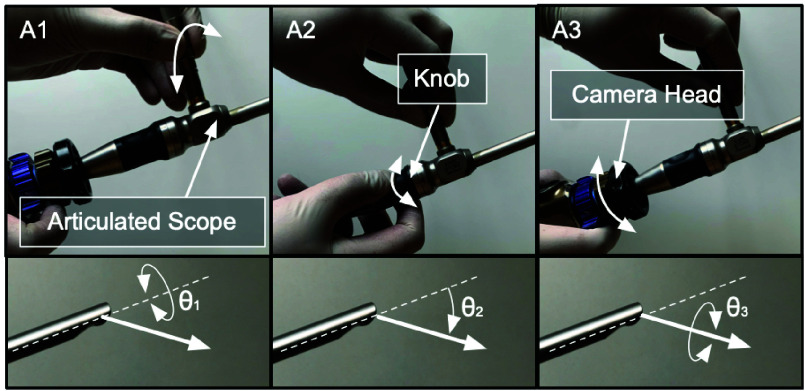
Manipulation of a variable-view rigid scope to modify the viewing direction during a diagnostic procedure. Panel A1, A2, and A3 shows the three distinct maneuvers performed by the operator to alter the viewing direction.

**FIGURE 2.**
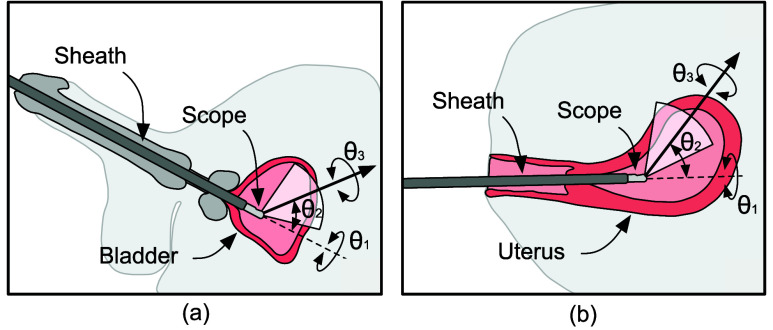
Schematic representation of variable-view rigid scope used in diagnostic procedures: (a) Rigid cystoscopy, and (b) Rigid hysteroscopy. In rigid cystoscopy, a scope is inserted into the urinary bladder via the urethra for diagnosis. In rigid hysteroscopy, a scope is inserted into uterine cavity through the cervix for diagnosis.

To perform a diagnostic procedure using a variable-view scope, it is inserted via a natural orifice and is manipulated using a combination of three distinct motions, all while keeping the position of the shaft fixed. These motions include: (a) rotation of the scope along its shaft to alter the viewing direction (Figure 1, panel A1), (b) turning of a knob located at the rear end of the scope to adjust the viewing-angle (Figure 1, panel A2), and (c) rotation of the camera head to adjust the orientation of the view displayed on the visualization screen (Figure 1, panel A3). For example, in a cystoscopy procedure (depicted in Figure 2a), a 0°-30° viewing-angle can be used to inspect the bladder’s base, posterior, and sidewalls; ~70° to inspect the bladder’s anterolateral wall and dome; and ~120° to inspect the anterior region of the bladder neck [9], [10]. Similarly, in a hysteroscopy procedure (depicted in Figure 2b), viewing angles can be altered from 0° to 120° to inspect the fundus, body, and cervix regions of the uterus [11].

**FIGURE 3.**
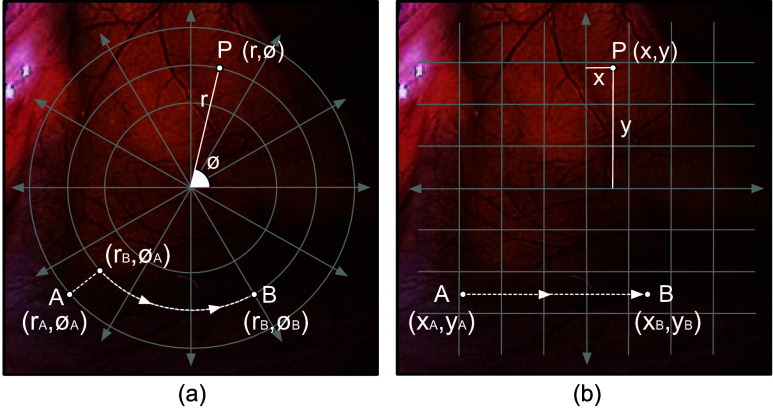
(a) Polar coordinate system mapped to a diagnostic site. (b) Cartesian coordinate system mapped to a diagnostic site.

Despite the advantage of a variable-view scope, it is challenging for an operator to effectively manipulate the scope. This is because of two primary reasons. First, the three coaxial motions cause alteration of the view within a polar co-ordinate system. As shown in Figure 3a, any point *P* on the operative field that needs to be visualized, can be characterized by a distance *r* from a reference point, and an angle $\phi $ from a reference direction. Adjusting the viewing angle and viewing direction results in modification to *r* and $\phi $. While transitioning from point *A* to point *B*, the operator must mentally compute a path and then modify *r* and $\phi $, accordingly. This spatial mapping increases the cognitive workload for the operator. Second, in addition to the spatial mapping, it is difficult for the operator to manually control a variable-view rigid scope. Operator’s one hand is occupied with holding the camera head and scope, while the other hand is required for rotating the knob and the scope shaft.

To address the aforementioned challenges, this work introduces a mechanized arm to securely hold a variable-view rigid scope, coupled with an actuation mechanism. The integrated system aims to provide an intuitive means to navigate the field-of-view during diagnostic procedures.

## II. Related Works

Existing robotic scope holders, such as EMARO [12], RoboLens [13], SoloAssist [14], FreeHand [15], and HIWIN MTG-H100 [16], offer an actuation mechanism to maneuver a fixed-angle rigid scope. These scope holders facilitate the pan (left-right), tilt (up-down), roll (shaft rotation), and zoom (insertion-retraction) motions of the scope through the incision point. Though these motions are essential for laparoscopic (keyhole) surgeries, they are not required during diagnostic procedures. The inclusion of additional actuation equipment for these motions contributes to a higher overall cost of the system. Moreover, the increased degrees-of-freedom exhibited by these systems demand more workspace during setup and usage in the operating room.

Unlike laparoscopic surgeries, diagnostic procedures require the scope shaft to remain stationary. This is necessary to prevent irritation to the natural opening (through which the scope is inserted) and minimize potential patient injury, or post-procedure discomfort. Thus, a more practical and cost-effective solution would involve (a) usage of a passive mechanical arm to support the weight of the variable-view scope and attached cables, and (b) incorporation of an actuation mechanism to maneuver the scope (as depicted in Figure 1) without moving the scope shaft. This would eliminate the need for unnecessary motions aligning with the specific requirements of diagnostic procedures, and would offer a cost-effective alternative to existing robotic scope holders.

Apart from robotic scope holders, several research prototypes of scopes with an inbuilt actuation mechanism have also been developed. References [17], [18], [19]. These scopes consist of a rigid shaft that transitions into a flexible distal end, enabling changes in the viewing direction. Legrand et al. [17] introduced the PliENT system, specifically designed to visualize nasal cavity pathologies without resecting healthy tissues. Another nasal system, presented by Song et al. [18], featured a custom designed endoscope incorporating a flexible bending section linked to a rigid shaft. Similarly, Li et al. [19] introduced a custom designed cardioscope consisting of a rigid shaft with adjustable length and angulation of the flexible section.

While these prototypes offer an increased field of view similar to variable-view rigid scopes, there are potential clinical drawbacks associated with the bending section. For example, during diagnostic procedure within confined spaces, there may be insufficient room for the flexible tip to bend. As the distal end bends and sweeps a region, it may contact the tissue surface within the cavity. This inadvertent interaction could harm the tissue or damage the scope components [20]. Moreover, the flexible distal end may also affect the stability of the view acquired from the scope [21]. Even after the diagnostic procedure, it is difficult to sterilize the complete system due to presence of electromechanical actuation components within the scope itself [22]. In contrast, a variable-view scope has a rigid distal tip. The variable viewing angle in the scope relies on a purely mechanical system (incorporating a rotating optical prism at the distal end). Since a bending mechanism and electromechanical components are absent, a variable-view scope is more robust and practical for diagnostic procedures. It minimizes the risk of damaging the scope during diagnosis in confined-spaces and simplifies the sterilization process.

## III. Methodology

### A. System Architecture

**FIGURE 4.**
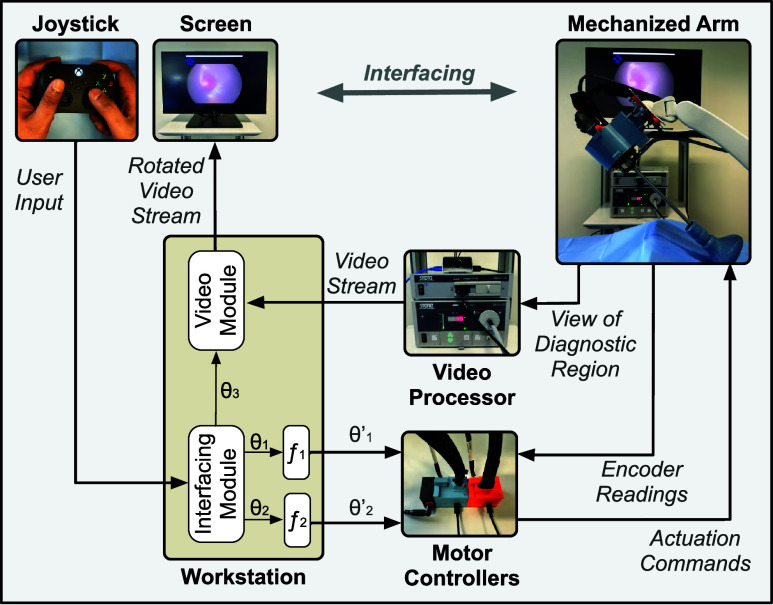
Architecture of the developed system to maneuver a variable-view rigid scope during a diagnostic procedure. The operator interacts with the system using a joystick. A mechanized arm is used to maneuver variable-view rigid scope to visualize different regions of the diagnostic site. The view of the diagnostic site acquired from the scope is processed and rendered back to the operator on a display screen.

The architecture of the developed system is presented in Figure 4. The operator uses a joystick (Xbox controller - Microsoft) to provide input commands to the system. The received input commands are processed on a workstation. On the workstation, two software modules (interfacing module and video module) run in parallel to process the information fetched from the hardware units. The interfacing module computes the required rotation of the scope shaft and the knob, and sends it to the motor controllers. The motor controllers further send the actuation commands to the mechanized arm for rotating the scope’s shaft and the knob. Simultaneously, the view of the diagnostic region acquired from the scope is fetched by the video processor. The video stream from the video processor is received by the video module running on the workstation. The video module applies software rotation to the video stream based on the angle computed by the interfacing module. The rotation ensures the scope’s horizon is maintained during the diagnostic procedure. The rotated video stream is then rendered back to the operator on a display screen.

### B. Mechanized Arm

**FIGURE 5.**
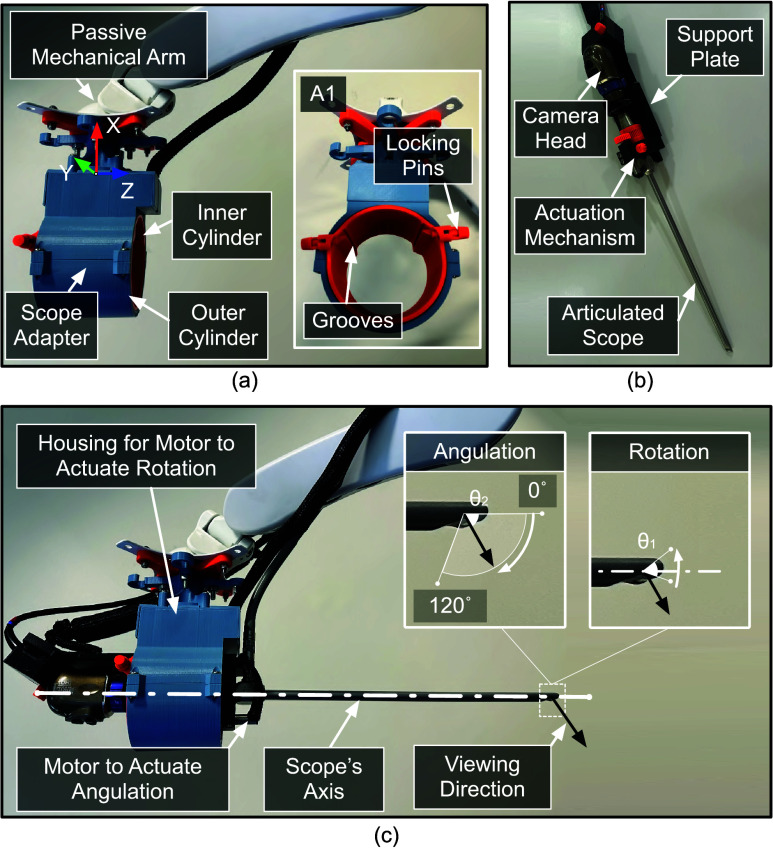
(a) Mechanized arm for supporting and actuating a variable-view rigid scope. (b) Support plate hosting the scope with camera head. (c) Change in the viewing direction of the scope produced by the actuation of the motors.

The mechanized arm comprises of a scope adapter suspended from a passive mechanical arm (Figure 5a). The scope adapter consists of two concentric cylinders, where the inner cylinder rotates with respect to the outer cylinder. The design details are presented in our previous work [23], [24]. The inner cylinder consists of grooves in which a support plate can be inserted and locked into position using locking pins (Panel A1 of Figure 5a). A support plate is used to host the camera head and the scope (Figure 5b). It also consists of an actuation mechanism to rotate the knob at the rear end of the scope. Rotating the knob enables the scope’s viewing direction to change from 0° to 120° (Figure 5c). In addition, rotating the inner cylinder (hosting the support plate) results in scope and camera head rotating together along the scope’s axis. This causes the viewing direction to rotate along the scope’s axis in both clockwise and anticlockwise direction. (Figure 5c).

### C. User Interfacing

**FIGURE 6.**
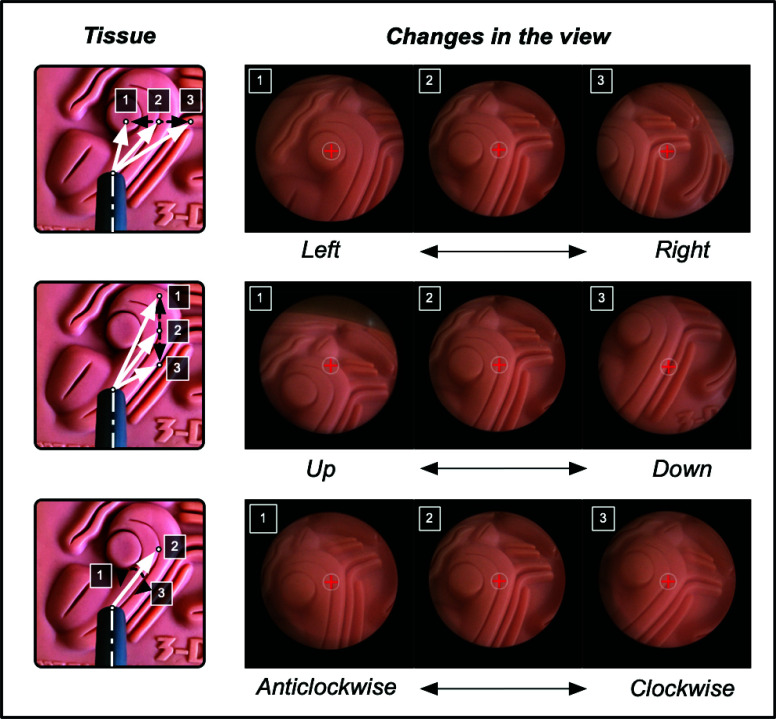
Input from the joystick is mapped to a specific movement of the viewing direction to visualize the tissue. Variation in the viewing direction changes the view acquired from the scope.

While a mechanized arm is able to securely hold and actuate a variable-view rigid scope, a spatial mapping is required that reduces the cognitive workload of the operator. A natural way to visualize different regions during a diagnostic procedure would be an actuation mechanism that enables the operator to maneuver the view within a Cartesian space. As shown in Figure 3b, a point *P* is defined by the distances *x* and *y* from two orthogonal coordinate axes. For an operator, it is more intuitive to move from point *A* to point *B* by adjusting the *x* and *y* parameters as compared to polar coordinate system (Figure 3a). This capability allows the operator to mentally map the surroundings and effortlessly shift the focus of the scope to the desired position for diagnosis. To align with this, an actuation mechanism was developed so that each actuation command alters the view in the Cartesian space (Figure 6).

A rotational frame was computed based on the input provided by the operator through the joystick. Each command caused the rotational frame to roll, yaw, and pitch along its axes. The rotational frame was processed to compute the following: (a) the rotation of the scope shaft represented by angle $\theta _{1}$, (b) the angulation of the scope viewing direction represented by angle $\theta _{2}$, and (c) the software-enabled rotation of the scope’s view rendered on the display represented by angle $\theta _{3}$. As the scope and the camera head were affixed on the support plate, the software enabled rotation on the screen produced the same effect as of rotating the scope with respect to the camera head.

The axes of the rotational frame were represented by unit vector $R_{X}$, $R_{Y}$, and $R_{Z}$. $R_{Z}$ and $R_{Y}$ were aligned to scope’s viewing direction and horizon requested by the operator, respectively. An orientation of a world coordinate frame (represented by X, Y, and Z axes) was defined as follows: X and Y axes were parallel to the scope rotational motor housing and the Z-axis was along the direction of the scope shaft (shown in Figure 5a). $\theta _{1}$ was computed as an angle substituted by a vector, which is orthogonal to the $R_{Z}$ vector and Z-axis, with respect to the Y-axis. $\theta _{2}$ was computed as the angle from the Z-axis to the $R_{Z}$ vector. $\theta _{3}$ was found to be the angle from the Y-axis of a new frame to the $R_{Y}$ vector. The new frame is computed by rotating an identity matrix along the Z-axis by $\theta _{1}$ and then along the Y-Axis by $\theta _{2}$.

**FIGURE 7.**
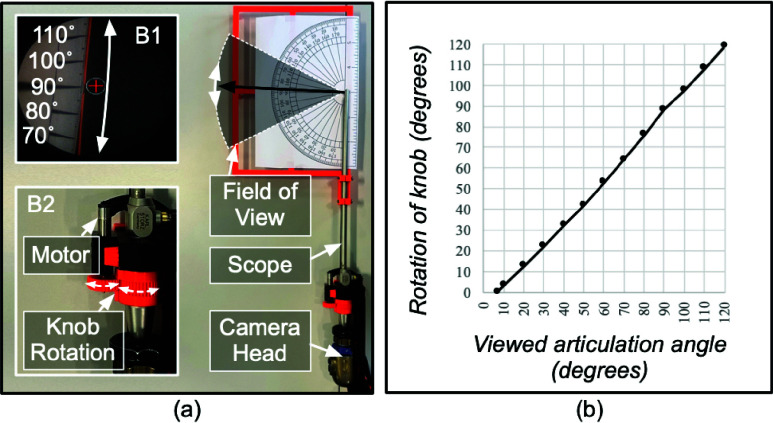
(a) Calibration setup to map the knob rotation performed by actuating the motor with the angle viewed by the scope. Panel B1 shows the view observed by the scope. Panel B2 shows the gear system. (b) Plot of the transfer function.

Before sending $\theta _{2}$ to angulate the scope’s viewing direction, two additional steps were performed via a transfer function $f_{2}$ applied to $\theta _{2}$ (Figure 4). First, the input knob rotation did not linearly modify the articulation angle, thus, a calibration setup was used to correct the mapping (Figure 7). The numerical value of the knob’s rotation performed by actuating the motor and the corresponding angle viewed by the scope was recorded (shown in panel B1 of Figure 7a). Samples were taken at a regular interval of 10 degrees and the intermediate values were interpolated to compute the calibration transfer function (plot shown in Figure 7b). Second, the scope adapter uses a gear system to transfer the rotation motion from the motor to the angulation knob (shown in panel B2 of Figure 7a). To compensate for the backlash introduced by the gear system, an offset rotation was applied to the motor when the angulation knob rotation was reversed. Similarly, a transfer function $f_{1}$ was applied to $\theta _{1}$ to adjust the gear ratio before sending it to rotate the scope shaft.

### D. System Evaluation

User studies were conducted to assess the system in two scenarios (namely Scenario-A and Scenario-B). In both scenarios, the variable-view rigid scope was maneuvered using three modes: (a) *manual mode – without support*, where the subject directly manipulated the scope to facilitate visualization (b) *manual mode – with support*, where the subject manipulated the scope shaft inserted in a trocar suspended by a mechanical arm, and (c) *actuated mode*, where the subject employed the proposed system for visualization. Eleven subjects (aged between 23 years and 40 years) from the Department of Surgery, Hamad General Hospital of Hamad Medical Corporation participated in the user study. The participants included academic researchers (non-surgeons) involved in development of surgical technologies. The participants exhibited proficiency in maneuvering angulated and articulated scopes. They were able to maintain focus and optimal distance from the operative view, and manage the scope horizon. The study was approved by institutional review board ethical committee (Medical Research Center, Doha, Qatar, approval number MRC-01-20-087). To familiarize the subjects with the system, a preparatory session was conducted before the study’s initiation. This session spanned 20 to 30 minutes, during which the subjects demonstrated their ability to correlate scope movements with changes in the operative field view under both *manual mode* and *actuated mode*. A time range of 20 to 30 minutes was reported as each subject took different time to get acquainted to the usage of the system. This session also included the time taken to introduce the scenarios and tasks of the user study to the subjects and concluded when the user exhibited satisfaction in mapping the movement of the scope to the user input. The study utilized a randomized cross-over study protocol. The participants were randomly assigned into two groups, *manual mode – without support* (n = 6) and *actuated mode* (n = 5). The first group performed the experiments in *manual mode – without support* followed by *actuated mode* while the order was reversed for the second group. Both groups then performed the *manual mode – with support* after a time gap of 4 months. The protocol engaged same set of participants for all modes. Within each mode, the order in which the two scenarios were performed was assigned based on simple randomization.

**FIGURE 8.**
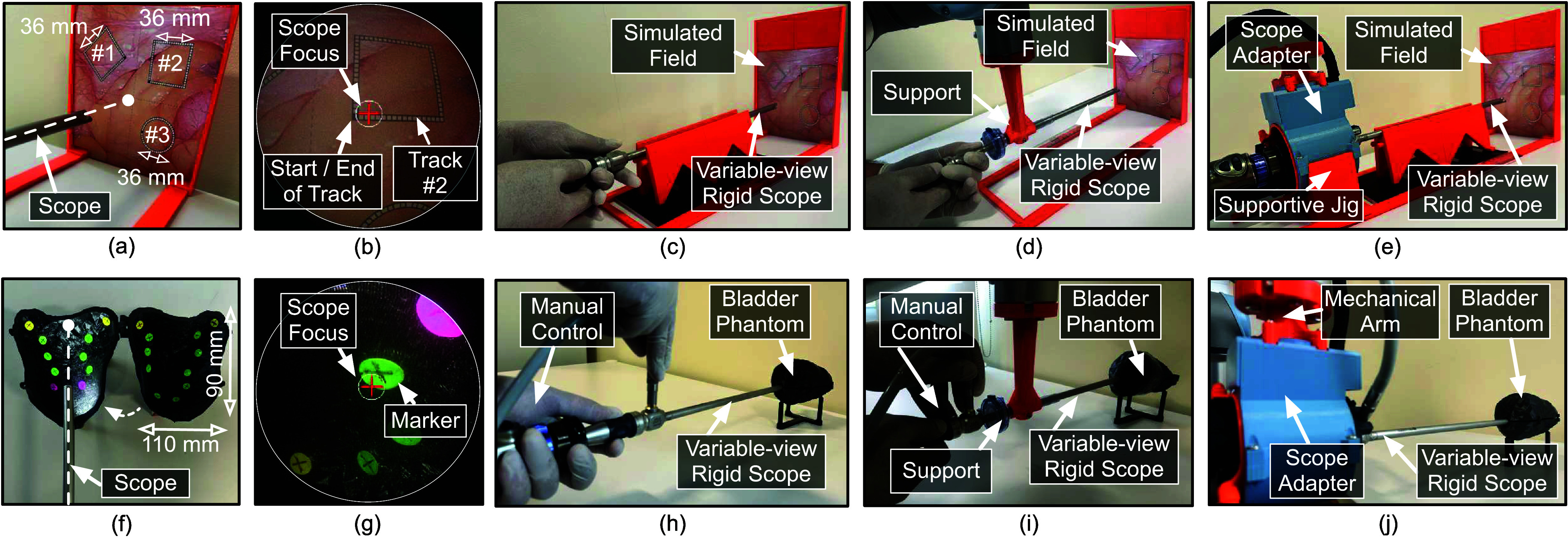
(a) Simulated diagnostic site of the user-study conducted in Scenario-A. (b) View acquired from the scope during traversal of track #2 in Scenario-A. (c) User study performed under manual mode without support in Scenario-A. (d) User study performed under manual mode with support in Scenario-A. (e) User study performed under actuated mode in Scenario-A. The scope adapter is placed on a supportive jig. (f) 3D printed urinary bladder illustrating the positioning of the markers along the bladder lining in Scenario-B. (g) View acquired from the scope while targeting a marker in Scenario-B. (h) User study performed under manual mode without support in Scenario-B. (i) User study performed under manual mode with support in Scenario-B (j) User study performed under actuated mode in Scenario-B. The scope adapter is suspended from a passive mechanical arm for Scenario-B.

Scenario-A evaluated the accuracy of the system in adjusting the scope focus to follow a predetermined path (Figure 8). Three tracks (#1, #2, and #3) were drawn on simulated operative field placed orthogonal to the scope (Figure 8a). The tracks were composed of multiple square segments, each measuring $2\times 2$ mm. The scope’s focus was rendered as a circled ‘+’ symbol on the simulated operative field view. This task required actuating the system to traverse the tracks from start to end, while ensuring the scope’s focus stays within the track borders (Figure 8b). Three parameters were recorded as response variables to assess the system’s performance under *manual mode – without support* (Figure 8c), *manual mode – with support* (Figure 8d) and *actuated mode* (Figure 8e): (a) duration to traverse the track, (b) percentage of time for which the scope’s focus deviates outside the track, and (c) number of missed segments while traversing the track.

Scenario-B assessed the feasibility of the system to explore and visualize an enclosed diagnostic area (Figure 8). A rigid cystoscopy procedure was simulated using a 3D-printed urinary bladder. Polyps were represented as 22 circular colored markers attached along the bladder lining [25]. As shown in Figure 8f, each marker was 8 mm in diameter and had a ‘+’ symbol. The task involved sequentially visualizing these markers by aligning scope’s focus (rendered as circled ‘+’ symbol) onto them (Figure 8g). The subjects proceeded to the next marker only when the preceding marker was accurately visualized. This was confirmed by an external observer during the task. The markers were positioned at the bladder’s posterior, anterior (anterosuperior), and lateral walls (left and right), positions that necessitated both scope rotation and angulation. In addition, software rotation of the view was performed to align the ‘+’ symbol of the scope’s focus with that of the markers. Thus, all three degrees-of-freedom were used to explore the enclosed diagnostic area. The setups in *manual mode – without support, manual mode – with support* and *actuated mode* are shown in Figures 8h, 8i and 8j. The duration to complete the task was recorded as a response variable. After concluding scenario-B, the subjects proceeded to fill out a NASA-TLX questionnaire, rating the mental, physical, temporal, performance, effort, and frustration aspects of the three modes on a scale ranging from 1 to 10. A lower rating by the subject represented a better evaluation for the corresponding aspect.

### E. Data Analysis

In this study, all subjects participated in each scenario-mode combination, and so for each subject one data point per scenario per mode was recorded. Paired *t*-tests were used to control the subject-to-subject variation when comparing the average performance across the different pair of responses. Scenario-A compared the three pairs of modes for each track, whereas Scenario-B and NASA-TLX scores compared the three pairs of modes. Since in each response variable we compared three modes, all possible comparisons result three possible pairs that are examined. To account for multiple comparison issues, in all aforementioned tests, we use a Bonferroni adjusted level of significance. Precisely, the cutoff value for determining significance is set to 0.0167 (equivalent to 0.05/3) to account for the three simultaneously performed hypothesis testing.

## IV. Results

**FIGURE 9.**
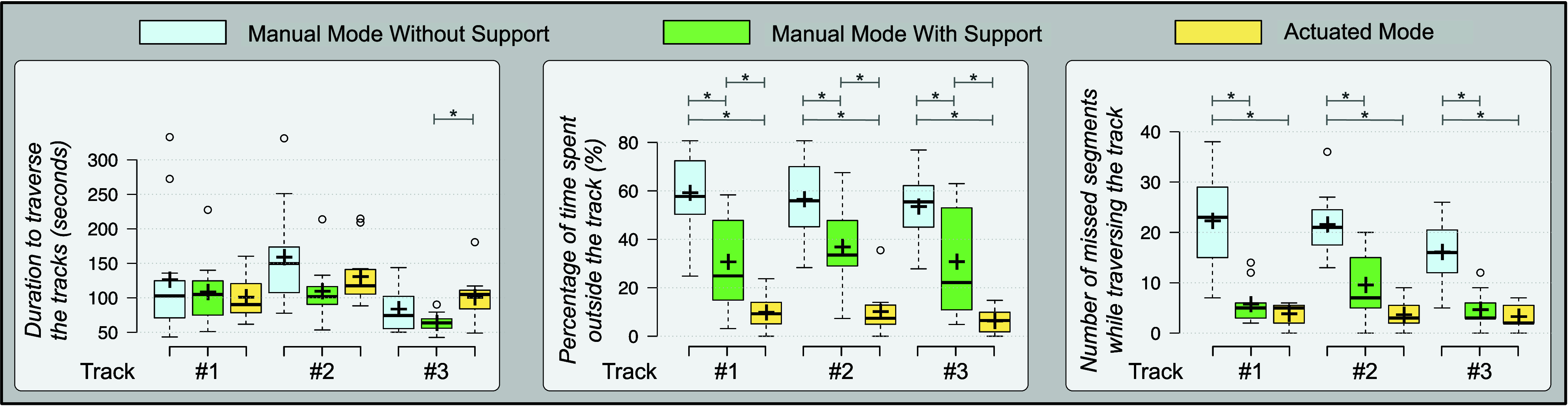
Boxplots presenting the measured parameters to assess the user study performed in Scenario-A. ‘*’ denotes a *p*-value less than 0.0167.

The results of the user-study for Scenario-A is presented in Figure 9. The duration to traverse the three tracks did not have any significant difference between the three modes except between *manual mode – with support* ( $63.5~\pm ~13.6$ s) and *actuated mode* ( $100.6~\pm ~33.0$ s, ${p} =0.01$) for track #3. However, the percentage of duration spent outside the tracks was significantly different between all three modes. In *manual mode – without support*, the scope’s focus deviated outside tracks #1, #2, and #3 for $59.2~\pm ~15.9$%, $56.5~\pm ~16.0$%, and $53.5~\pm ~13.1$% of the time, respectively. This was significantly reduced for *manual mode – with support* to $30.6~\pm ~19.2$% ( ${p} =0.005$), $36.8~\pm ~17.6$% ( ${p} =0.02$), and $30.7~\pm ~22.1$% ( ${p} =0.008$) respectively for tracks #1, #2, and #3. In addition, the *actuated mode* exhibited improved performance as compared to both the *manual modes* of operation, with a significantly lower time percentage outside tracks #1, #2, and #3 at $9.8~\pm ~6.6$% ( ${p} < 0.001$ for manual – without support and ${p} =0.02$ for manual – with support), $10.1~\pm ~9.3$% ( ${p}~< 0.001$ for manual – without support and ${p} =0.004$ for manual – with support), and $6.2~\pm ~4.9$% ( ${p} < 0.001$ for manual – without support and ${p} =0.01$ for manual – with support), respectively. Similarly, the *manual mode – with support* exhibited better results for number of missed segments in comparison to *manual mode – without support*. For tracks #1, #2 and #3, the number reduced from $22.3~\pm ~9.6$, $21.5~\pm ~6.1$, and $16.1~\pm ~6.5$ to $5.7~\pm ~3.7$ ( ${p} < 0.001$), $9.5~\pm ~6.1$ ( ${p} =0.002$), and $4.6~\pm ~3.3$ ( ${p} < 0.001$). Likewise, the *actuated mode* performed significantly better than *manual mode – without support* in terms of number of missed segments for tracks #1, #2 and #3, reducing it to $3.8~\pm ~1.9$ ( ${p} < 0.001$), $3.6~\pm ~2.6$ ( ${p}~< 0.001$), and $3.3~\pm ~2.5$ ( ${p} < 0.001$). There was no significant difference between *actuated mode* and *manual mode – with support* for the number of missed segments.

**FIGURE 10.**
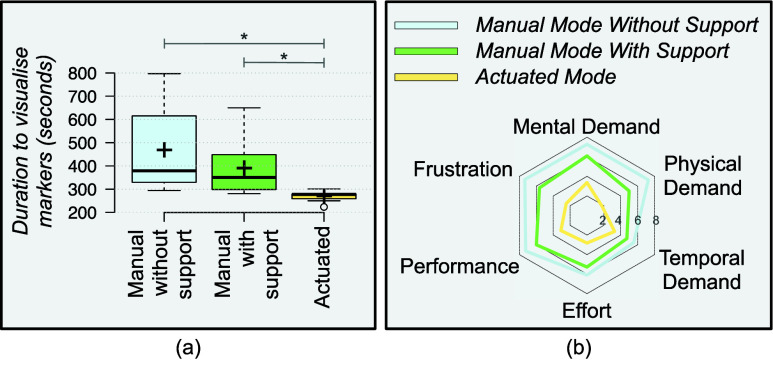
(a) Boxplot presenting the total task duration in Scenario-B. ‘*’ denotes a *p*-value less than 0.0167. (b) The average scores using the NASA-TXL workload assessment scale (from 1 to 10) in Scenario-B for all modes.

The results of the user-study conducted in Scenario-B is presented in Figure 10a. The total duration to align the scope’s focus on each target was least for the *actuated mode*, followed by *manual mode – with support* and then *manual mode – without support*. The overall time taken to complete the task using *manual – without support* navigation ( $468.6~\pm ~165.4$ seconds) was almost double the time taken by the *actuated mode* ( $269.8~\pm ~20.6$ seconds, ${p} < 0.001$). The time taken to complete the task in *manual mode – with support* ( $390.5~\pm ~111.5$ seconds) was also significantly higher than the *actuated mode* ( ${p} =0.004$). There was no statistical significance between the two *manual modes*. Thus, the mechanism used in the *actuated mode* reduced subject effort when shifting scope focus to desired target positions.

The NASA-TLX scores (shown in Figure 10b) also indicated a lower cognitive workload while operating in *actuated mode* as compared to *manual modes*. Under *manual mode – without support*, the NASA-TLX scores were $7.3~\pm ~1.5$ for mental demand, $7.3~\pm ~1.3$ for physical demand, $5.5~\pm ~1.7$ for temporal demand, $7.3~\pm ~2.3$ for effort, $6.1~\pm ~2.2$ for performance, and $7.4~\pm ~2.7$ for frustration. Under *manual mode - with support*, the NASA-TLX scores were $6.1~\pm ~1.9$ for mental demand, $5.0~\pm ~1.4$ for physical demand, $4.7~\pm ~1.5$ for temporal demand, $6.0~\pm ~2.3$ for effort, $5.3~\pm ~2.3$ for performance, and $5.6~\pm ~2.7$ for frustration. Whereas the scores under *actuated mode* were $3.4~\pm ~1.6$ for mental demand, $1.8~\pm ~0.8$ for physical demand, $3.3~\pm ~1.2$ for temporal demand, $3.1~\pm ~1.4$ for effort, $2.8~\pm ~1.6$ for performance and $2.5~\pm ~1.2$ for frustration. There was a statistical significance (p <0.001) between the *manual modes* and *actuated mode* for all the parameters except temporal demand. The subjects were able to mentally map the surroundings and issue a corresponding actuation command via the user interface.

## V. Discussion

The proposed mechanized arm significantly improved an operator’s ability to accurately explore the diagnostic site, effectively addressing the challenges encountered in *manual modes*. First, under *manual modes*, the subjects struggled to maintain a clear sense of the scope’s horizon (particularly within the bladder cavity). As a result, the variable-view rigid scope was inadvertently moved in undesired directions. This issue was successfully resolved in *actuated mode*. A Cartesian space was mapped onto the view of the diagnostic site rendered on the screen and the subjects were able to issue actuation commands within this space. A direct one-to-one mapping existed between the direction the subject intended to move the scope’s focus (via input through joystick) and the resulting change in the view (produced by the actuation of the variable-view rigid scope). Second, the *manual modes* required subjects to perform three coaxial maneuvers (camera head rotation, scope shaft rotation, and knob rotation along the same axis), leading to an increase in the cognitive workload. In *actuated mode*, these maneuvers were performed by the mechanized arm and were transparent to the subjects who issued commands to alter the view in Cartesian space. Lastly, due to the weight of the system (comprising of the variable-view rigid scope, camera head, and attachments to light source), it was difficult to hold the scope steadily during the diagnostic procedure in *manual mode – without support*. During the user-study, this posed challenges in completing the tasks as one hand was engaged in holding the camera head while the other hand was used to manipulate both the knob and the shaft. Additionally, the light source cable attached to the scope could not stay upright due to the weight and had to be continuously kept in place to avoid unintentional shaft rotation. Although, *manual mode – with support* relieved the users from holding the weight of the camera head and scope, the light source cable issue remained persistent. In *actuated mode*, these issues were overcome by incorporating a mechanical arm to suspend the scope along with camera head and enclosing the light source cable within the support plate.

There were several benefits which the *actuated mode* inherited. First, the viewing angle was controlled at a higher precision (of 0.5°) by actuating the knob of the variable-view rigid scope using a motor. However, in *manual modes* the operator could only secure the knob at discrete orientations (of 0°, 30°, 45°, 60°, 90°, and 120°). Second, in *actuated mode*, the scope adapter was supported by a jig or suspended from a passive mechanical arm, which ensured the position of the rigid scope shaft was stable during the diagnosis. Unlike *manual mode – without support*, the support prevented tilting/panning of the scope shaft. This will prevent irritation or injury to the tissue during diagnosis [26], [27].

It should be noted that both rigid and flexible scopes are utilized in clinical scenarios such as cystoscopy [25], with no definitive assurance that flexible endoscopy offers greater comfort compared to its rigid counterpart [28]. In the context of hysteroscopy, while flexible hysteroscopy is noted for its reduced pain throughout the procedure, rigid hysteroscopy excels in terms of visibility, visualization, and diagnostic accuracy [29]. Moreover, specific procedures conducted using rigid endoscopy necessitate the utilization of multiple scopes of different angles, making the use of a variable-view rigid scope advantageous. For instance, the maxillary antrostomy procedure is initiated with a 0° scope followed by multiple angled scopes (30°, 45°, and 70°) to visualize different areas of the nasal cavity [30]. The repetitive insertion and removal of different angled scopes adds to the procedure time. Thus, the problem addressed in this study remains pertinent, and the proposed mechanized arm tends to alleviate the challenges faced by the operator in maneuvering a scope for diagnosis.

This study has certain limitations. First, a 10-mm diameter variable-view scope was used in the user-study to simulate the cystoscopy procedure, in contrast to the standard 4-mm scope [31]. Despite the use of a larger diameter scope, the developed actuation mechanism is applicable for smaller diameters scopes, necessitating only the recalibration of the transfer function and offset rotation. Secondly, this work represents a preclinical study with a limited sample size (n = 11), conducted to evaluate the capability of the developed actuation mechanism in diagnostic procedures. To substantiate the benefits across diverse diagnostic scenarios, additional randomized controlled trials involving a larger sample size with varying levels of surgical expertise and different variable-view rigid scopes are needed.

The proposed system was designed for ease in translation to the clinical practice. The system provides intuitive mechanism to control an articulated scope. This would improve the spatial understanding of the diagnostic region to be visualized, thereby promising enhanced results in clinical usage. In addition, due to its modular design, the system can be easily modified to be fitted to any bedside mechanical arm that is already available. Finally, the system set-up essentially consists of the same steps as for conventional mode with the inclusion of only two additional steps: enclosing scope in the support plate and inserting it into scope adapter.

The current study also enhances the control mechanism in comparison to our prior works [23], [24]. The prior works uses one-to-one mapping between a user input and a scope movement (shaft rotation, lens angulation, or software rotation). While this is suitable to visualize around a periphery of a circular section (for example periphery of a circular disk [23] or lung boundaries [24]), it not feasible to visualize the area interior to the circular section. The precise angles needed to move in a straight line (horizontally or vertically) across the circular section is difficult for the operator to mentally compute and execute. In this study, the proposed Cartesian based control mechanism overcomes this problem and allows operator to intuitively explore the complete diagnostic area.

The proposed system, apart from diagnostic procedures, holds potential for application in Minimally Invasive Surgery (MIS) and Natural Orifice Transluminal Endoscopic Surgery (NOTES). Instead of using a mechanical arm, the scope adapter can be attached to a robotic arm with six degrees-of-freedom. This will enable the scope shaft to be panned, titled, and inserted/retracted along the incision point to visualize the insufflated cavity during a MIS. It would require designing new human-computer-interfacing mechanism for operating surgeon to provide actuation commands and the system to process them [32]. In case of NOTES, the support plate can be modified to actuate flexible endoscopes distal end [33].

It should be noted, as the proposed system was tested on a simulated clinical scenario, it stands at a *Technology Readiness Level* (TLR) of 5 and is pre-clinical research on a translational science spectrum. To further advance it to TRL 6, animal studies would need to be conducted. It is expected that the outcomes of the animal study will align with those of the conducted bench study. As a part of future work, we plan to (a) modify the design of the scope adapter to hold additional instruments (such as sheath and bridge in case of rigid cystoscopy), (b) integrate image mosaicing techniques to map the surroundings into a three-dimensional virtual model of the cavity for better visualization, and (c) assess the potential of using hands-free user interfaces (such as devices based on head-motion and eye-gaze) for scope manipulation. Modular structure of the proposed system facilitates easy modification of the mechanized arm, enabling the incorporation of extra holders for surgical tools like grasping forceps, cautery, or loop diathermy. The inclusion of a control mechanism for the added loop diathermy will enhance the system’s capability to perform office-based procedures, such as transurethral resection of bladder tumors [34] and treatment of cervical dysplasia [35].

## VI. Conclusion

The work involves the implementation of a mechanized arm designed to enhance the maneuverability of a variable-view rigid scope during diagnostic procedures. It enables the operator to intuitively adjust the viewing direction of the scope for optimal visualization of the diagnostic site, thereby minimizing cognitive workload. Moreover, it effectively prevents unintended movements of the scope shaft without compromising on the field-of-view to visualize the complete diagnostic site. This, in turn, reduces the risk of potential injury to the natural openings and passages through which the scope is inserted into the patient during diagnosis.
